# Clinical Notes

**Published:** 1876-10

**Authors:** Julius A. Post


ART. II.—*Clinical Notes.* By JULIUS A. POST, M. D.

On the fifteenth of September 1875, Mr. B., aged 32 years, married, by occupation a newspaper reporter, called at my office and gave me the following history. About a year ago he began to loose the faculty of complete sexual intercourse, followed by involuntary seminal emissions and great irritability of the urethra, and now presented the appearance of a man whose nervous system was completely exhausted. He had been treated by several medical men without benefit, and had given his case up as hopeless. I persuaded him to make another trial. He assured me that masturbation had nothing to do with the case, as he never was addicted to the practice. His bowels were constipated, appetite poor, sleeps badly, and his whole nervous and general system greatly debilitated. Chemically, his urine presented nothing abnormal; microscopically, showed abundance of uric acid crystals, and occasionally, in the morning, a few stray spermatozoa. He was ordered senna confection at night sufficient to move the bowels. Quinn. sulph. grs. ii, with Ferri. Pulv. grs. v, in wine after each meal, and Acid Muriatic dil. gtts. ii, in simple bitters before eating, with warm bath at bedtime and an injection, per urethram, of warm water ℥i and Plumbi Acetas grs. ii, in solution several times a day; this he followed faithfully for two weeks, at the end of that time his general appearance had improved somewhat, but the sexual apparatus remained *in statu quo*, and he began to get impatient as to the result. The bitters and acid discontinued, and ordered Acid Phosphoric dil. gtts. v, with Tinct. Nux Vom. gtts. ii before meals in wine, and began to introduce a gum catheter, cooled with ice, to as low a temperature as he conld bear, three times a day. He thought he felt less irritability of the urethram, but soon grew discouraged, and in order to hold him I was obliged to change the treatment in some way. His general condition had improved very much; appetite good, bowels regular and rested well nights. I determined to continue the remedies as before and try electricity, using the Galvano-Faradic apparatus and employing the interrupted current of low intensity, using my hands as electrodes, and confining the action to the genitals and rectum. This I began to use three times a day. It was followed by a marked improvement, and in three weeks time he reported himself in good condition for satisfactory duty, and has continued so ever since. The improvement seemed to be due to the electricity.

